# *In vitro* analysis of the cytotoxic and anti-inflammatory effects of antioxidant compounds used as additives in ultra high-molecular weight polyethylene in total joint replacement components

**DOI:** 10.1002/jbm.b.32798

**Published:** 2012-08-22

**Authors:** C L Bladen, L Tzu-Yin, J Fisher, J L Tipper

**Affiliations:** Department of Biological Sciences, Institute of Medical and Biological Engineering, University of LeedsLS29JT, UK

**Keywords:** cytotoxicity, antioxidant, anti-inflammatory

## Abstract

Ultra high-molecular weight polyethylene (UHMWPE) remains the most commonly used material in modern joint replacement prostheses. However, UHMWPE wear particles, formed as the bearing articulates, are one of the main factors leading to joint replacement failure via the induction of osteolysis and subsequent aseptic loosening. Previous studies have shown that the addition of antioxidants such as vitamin E to UHMWPE can improve wear resistance of the polymer and reduce oxidative fatigue. However, little is known regarding the biological consequences of such antioxidant chemicals. This study investigated the cytotoxic and anti-inflammatory effects of a variety of antioxidant compounds currently being tested experimentally for use in hip and knee prostheses, including nitroxides, hindered phenols, and lanthanides on U937 human histocyte cells and human peripheral blood mononuclear cells (PBMNCs) *in vitro*. After addition of the compounds, cell viability was determined by dose response cytotoxicity studies. Anti-inflammatory effects were determined by quantitation of TNF-α release in lipopolysaccharide (LPS)-stimulated cells. This study has shown that many of these compounds were cytotoxic to U937 cells and PBMNCs, at relatively low concentrations (micromolar), specifically the hindered phenol 3,5-di-tert-butyl-4-hydroxyhydrocinnamate (HPAO1), and the nitroxide 2,2,6,6-Tetramethylpiperidine 1-oxyl (TEMPO). Lanthanides were only cytotoxic at very high concentrations and were well tolerated by the cells at lower concentrations. Cytotoxic compounds also showed reduced anti-inflammatory effects, particularly in PBMNCs. Careful consideration should therefore be given to the use of any of these compounds as potential additives to UHMWPE. © 2012 Wiley Periodicals, Inc. J Biomed Mater Res Part B: Appl Biomater, 101B: 407–413, 2013.

## INTRODUCTION

Total joint replacement (TJR) is a surgical procedure in which the natural joint is replaced with artificial components to alleviate pain and disability in late stage arthritis. In the United Kingdom, there are approximately 70,000 total hip replacements (THR) performed each year, and the lifespan of the replacement joint is 10–20 years. An ultra high-molecular weight polyethylene (UHMWPE) acetabular cup coupled with either a metal or ceramic femoral head are the gold standard materials for TJR. However, the lifespan of UHMWPE is compromised by material limitations and oxidative degradation resulting in increased wear rates.^1^ In addition, there is an increase in younger and more active patients requiring joint replacement and current UHMWPE materials lasting only 10–20 years are often out lived by the patient.

The development of novel formulations of UHMWPE to improve the mechanical properties and increase the lifespan of artificial joints is therefore essential. Gamma-irradiation sterilization in air causes oxidative degradation and delamination of UHMWPE.^1^, ^2^ Because of the limitations of UHMWPE wear and delamination, a new generation of highly crosslinked UHMWPE (HXLPE) was introduced, which decreased free radicals within the polymer as a result of post-irradiation melting techniques.^3^ However, this process has been shown to also reduce fatigue strength in the artificial joints, leading to concerns regarding fatigue crack propagation and rim failure.^4^, ^5^ Because of this limitation, alternative approaches have been sought to improve oxidative stability after radiation. For example, Vitamin E (α-Tocopherol) has been incorporated into UHMWPE. This process has been shown to increase wear resistance, toughness, and oxidative stability compared with HXLPEs.^6^ More recently, there have been increasing numbers of studies into the incorporation of different antioxidants into UHMWPE to improve oxidative resistance.

Vitamin E is a lipophilic antioxidant that can protect UHMWPE from oxidative degradation by eliminating free radicals.^7^ Vitamin E-doped irradiated UHMWPE can be manufactured by mixing Vitamin E with UHMWPE powder before consolidation, or by diffusing vitamin E into bulk UHMWPE.^8^ This novel material has shown improvements in wear resistance, bending fatigue resistance, and oxidative stability.^9^

Recent studies have investigated the addition of different chemicals to UHMWPE reduce oxidative degradation of the polyethylene. Nitroxides have been recognized as an oxidation stabilizer (antioxidant). Their methyl groups were able to quench the radical species and provide radioprotection in an *in vitro* study. An example of a nitroxide, 2,2,6,6-Tetramethylpiperidine 1-oxyl (TEMPO)-doped UHMWPE has been shown to quench alkyl free radicals.^10^ Hindered phenol antioxidants (HPAO), which possess stability against oxidative degradation, have been used in polymers and lubricants.^11^ HPAO1 [pentaerythritol tetrakis (3,5-di-tert-butyl-4-hydroxyhydrocinnamate)], HPAO2 and HPAO3 have been shown to provide oxidative resistance after 10 weeks of accelerated aging.^12^ HPAO1 and TEMPO both conferred protection against the toxicity of the solvents (DMSO, acetone, and ethanol), an effect shown previously for TEMPO and protection against oxidative damage caused by Cr ions in lymphocytes.^13^ Lanthanides are unique metal elements with applications in clinical diagnosis and have potential pharmaceutical value because of their dual biological effects.^14^ For instance, lanthanides can both promote and inhibit cell proliferation and apoptosis, and antioxidant activity and pro-oxidant activity depending on their concentrations (doses).^15^ Europium (II) and Europium (III) have shown to improve loading bearing capacity and reduce oxidation in UHMWPE.^16^l-glutathione is an important antioxidant synthesized in cells and protects cells from oxidative damage and maintains redox homeostasis.^17^ Also, glutathione reduction has been shown to lead to apoptosis.^18^ Menadione (Vitamin K3) is different from antioxidants. It is a chemotherapeutic agent and is known to induce cell death by oxidative stress.^19^, ^20^

The factors influencing TJR failure have been studied over two decades and evidence has shown that late aseptic loosening is the most significant problem.^21^ UHMWPE wear particles, generated from the UHMWPE acetabular cup articulating against a metal or ceramic femoral head, enter the periprosthetic tissues and are phagocytosed by macrophages. The macrophages then release pro-inflammatory cytokines that stimulate osteoclasts to resorb bone around the implant resulting in osteolysis and aseptic loosening. Inflammatory responses depend on particle size (0.1–1.0 μm) and volume (10–100 μm^3^ of particles/cell) stimulating inflammatory cytokines and chemokines, including tumor necrosis factor alpha (TNF-α), interlukin-1 beta (IL-β), IL-6, IL-8, and prostaglandin E2 (PGE2).^21^

Recent studies, have shown that vitamin E has the ability to down regulate the inflammatory response to UHMWPE wear particles.^22^, ^23^ Therefore, vitamin E, an antioxidant with additional anti-inflammatory effects, may have the potential to reduce osteolysis and further prevent TJR failures. However, based on the current literature, it remains to be established whether other antioxidants have the same effects as Vitamin E *in vitro*, and whether these chemicals are toxic to cells.

The aim of this study was to determine the cytotoxicity of several different antioxidant compounds in the monocyte like U937 cell line and in primary human mononuclear cells (PBMNCs), and to ascertain if any of the compounds were able to reduce the osteolytic potential of UHMWPE particles, which may lead to longer lasting TJR components that may be suitable for younger and more active patients. The effect of the addition of the antioxidant compounds on TNF-α production, an osteolytic cytokine, was monitored after lipopolysaccharide (LPS) stimulation for 24 h, thus, giving an indication of anti-inflammatory properties of the different compounds.

## MATERIALS AND METHODS

The antioxidant compounds and control compounds used in the study are listed in Table I.

**TABLE I:** Antioxidant Compounds Used in the Study

Compound Group	Antioxidant	Supplier
Natural antioxidant	Vitamin E	Merck
Nitroxide	TEMPO (2,2,6,6-tetramethylpiperidine 1-oxyl)	Sigma Aldrich Ltd
Hindered phenol	HPAO1 [pentaerythritol tetrakis (3,5-di-tert-butyl-4-hydroxyhydrocinnamate)]	Sigma Aldrich Ltd
Lanthanide	Europium II chloride and Europium III chloride	Sigma Aldrich Ltd
Quinone (positive control for oxidative stress induction)	Menadione (2-methyl-1,4-naphthoquinone)	Sigma Aldrich Ltd

### Cell culture

Human leukemic monoblast cells (European Collection of Cell Culture, ECACC), which expresses monocyte-like characteristics and can differentiate into macrophages, were maintained in RPMI 1640 medium supplemented with 10% (v/v) fetal bovine serum and antibiotics (penicillin and streptomycin 100 μg/ml). Cells were seeded into U-bottomed 96-well plates (Nunc, Rochester, NY) at a density of 1 × 10^4^ before addition of the antioxidant compounds.

### Isolation of primary human PBMNCs

Ethical approval for use of human volunteers was granted by University of Leeds, Faculty of Biological Sciences ethics committee. PBMNCs were isolated from whole heparinized blood from healthy volunteers using lymphoprep gradients (Axis-Shield, Norway) and centrifugation (800*g*, for 30 min). Mononulear cells were seeded into 96-well tissue culture plates at a seeding density of 1 × 10^4^ cells per well in RPMI 1640 medium supplemented with 10% (v/v) fetal bovine serum and antibiotics (penicillin and streptomycin 100 μg/ml). A minimum of three healthy donors were used for each experiment and were repeated a minimum of three times.

### Antioxidant stock solutions

Vitamin E was provided as dl-α-tocopherol acetate at a concentration of 500 mg/ml (Merck, Germany). A 20 mM stock solution of Vitamin E was prepared in RPMI 1640 cell culture medium and serial dilutions were made to achieve a working concentration (800 μM for Vitamin E). Stock solutions of 10 mM HPAO1 and 10 mM TEMPO were made and resuspended in ethanol (Table I). Stock solutions of 20 mM Eu(II) and 20 mM Eu (III) (Table I) were resuspended in RPMI 1640 cell culture medium, and serial dilutions were made to achieve working concentrations [25 μM for HPAO1, 3 μM for TEMPO, 75 μM for Eu (II) and (III)]. A wide range of doses from 0 μM to 5 or 10 mM were tested with both cell types. Controls included working concentrations of 1 mM l-glutathione and 75 μM Menadione.

### Cytotoxicity assays

Cell viability was assessed using the ATP-Lite™ assay (Perkin Elmer, Cambridge, UK) after 24 h. Results were expressed as mean counts ± 95% confidence limits and values were compared with the negative control using one-way ANOVA (*p* < 0.05) and Bonferroni post-test. LC50s (median lethal dose where 50% death occurs) were then calculated for each compound.

### LPS stimulation of either U937 cells or PBMNCs followed by addition of antioxidants

U937 cells or PBMNCs were stimulated with LPS at a concentration of 1000 ng.ml^−1^ for U937 cells and 200 ng.ml^−1^ for PBMNCs (concentrations calculated from prior in-house studies) for 3 h before the addition of antioxidant compounds. Antioxidants were then added at a non-cytotoxic dose to see if the antioxidant had an effect on the amount of TNF-α produced by the LPS-stimulated cells. Doses were as follows: Menadione, (positive control), 75 μM; Glutathione, (negative control), 1 mM; Vitamin E, 800 μM; HPAO1, 25 μM; TEMPO, 3 μM; Europium II and III, 75 μM. Doses used were determined by comprehensive in-house dose response/cytotoxicity studies to allow determination of sub-lethal doses where antioxidant activity was observed. Cells were incubated with antioxidants for 24 h at 37°C in an atmosphere of 5% (v/v) CO_2_ in air and each experiment was repeated a minimum of three times and with a minimum of three donors.

### ELISA

Cytokine production (TNF-α) was determined by ELISA (R and D systems, Cambridge, UK) after 24 h. Results were expressed as cytokine released in pg/ml ± 95% confidence limits and values were compared to the negative control using one-way ANOVA (*p* < 0.05) and Bonferroni post-test.

## RESULTS

### Cytotoxic effects of antioxidant compounds in U937 cells

**FIGURE 1:**
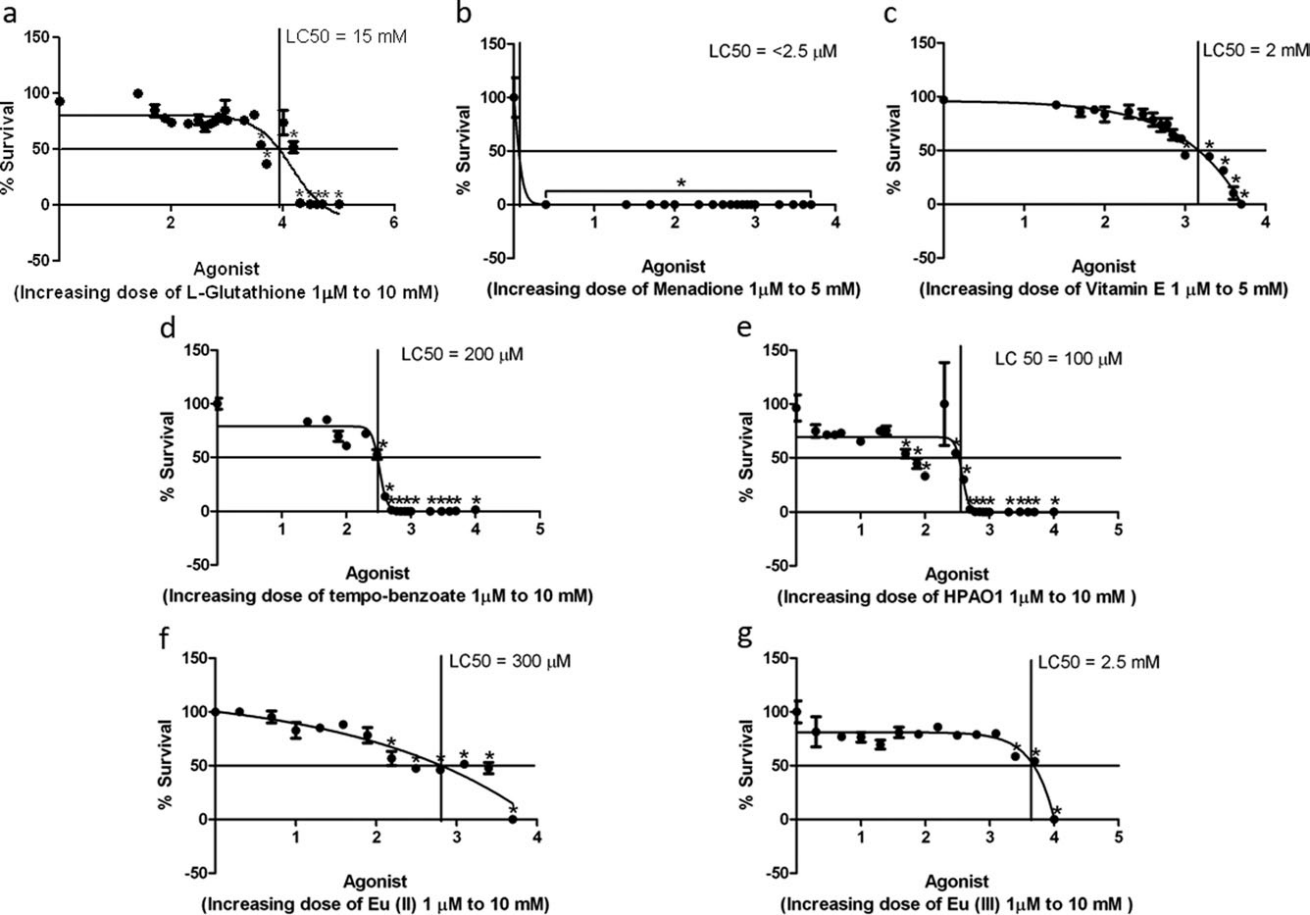
Dose response curves in U937 cells. The effect of increasing doses of l-Glutathione (panel a), Menadione (panel b), Vitamin E (panel c), Tempo (panel d), HPAO1 (panel e), Eu(II) (panel f), and Eu(III) (panel g) on U937 cell viability was investigated. Cell viability was determined using the ATPLite™ assay (Perkin-Elmer). Data were fitted to a sigmoidal dose response curve by log transforming the X column values then normalizing the Y column values. A non-linear regression was then performed before fitting the data to a sigmoidal dose response curve with a variable slope.

Negative (l-glutathione 1 mM) and positive (menadione 75 μM) controls were included in this study. In U937 cells, the LC50 for l-glutathione was 15 mM and for menadione was <2.5 μM [Figure 1(a,b)]. Vitamin E and europium (III) chloride (diluted in RPMI 1640 cell culture media) adversely affected cell viability only at relatively high concentrations (mM) in U937 cells: the LC50 values were 2 mM and 2.5 mM, respectively [Figure 1(c,g)]. TEMPO (diluted in ethanol), HPAO1, and Europium (II) chloride (diluted in cell culture media), adversely affected cells at relatively low doses, with their LC50 values being 200 μM, 100 μM, and 300 μM, respectively [Figure 1(d–f)]. A control for ethanol concentration was included and the cytotoxic point for ethanol in U937 cells was found to be at 5% (v/v) (data not shown). The cytotoxic doses of 100 μM (HPAO1) and 200 μM (TEMPO) contained ethanol concentrations of 0.8% (v/v) and 1.6% (v/v), respectively, well below the cytotoxic level.

### Cytotoxic effects of antioxidant compounds in PBMNCs

**FIGURE 2:**
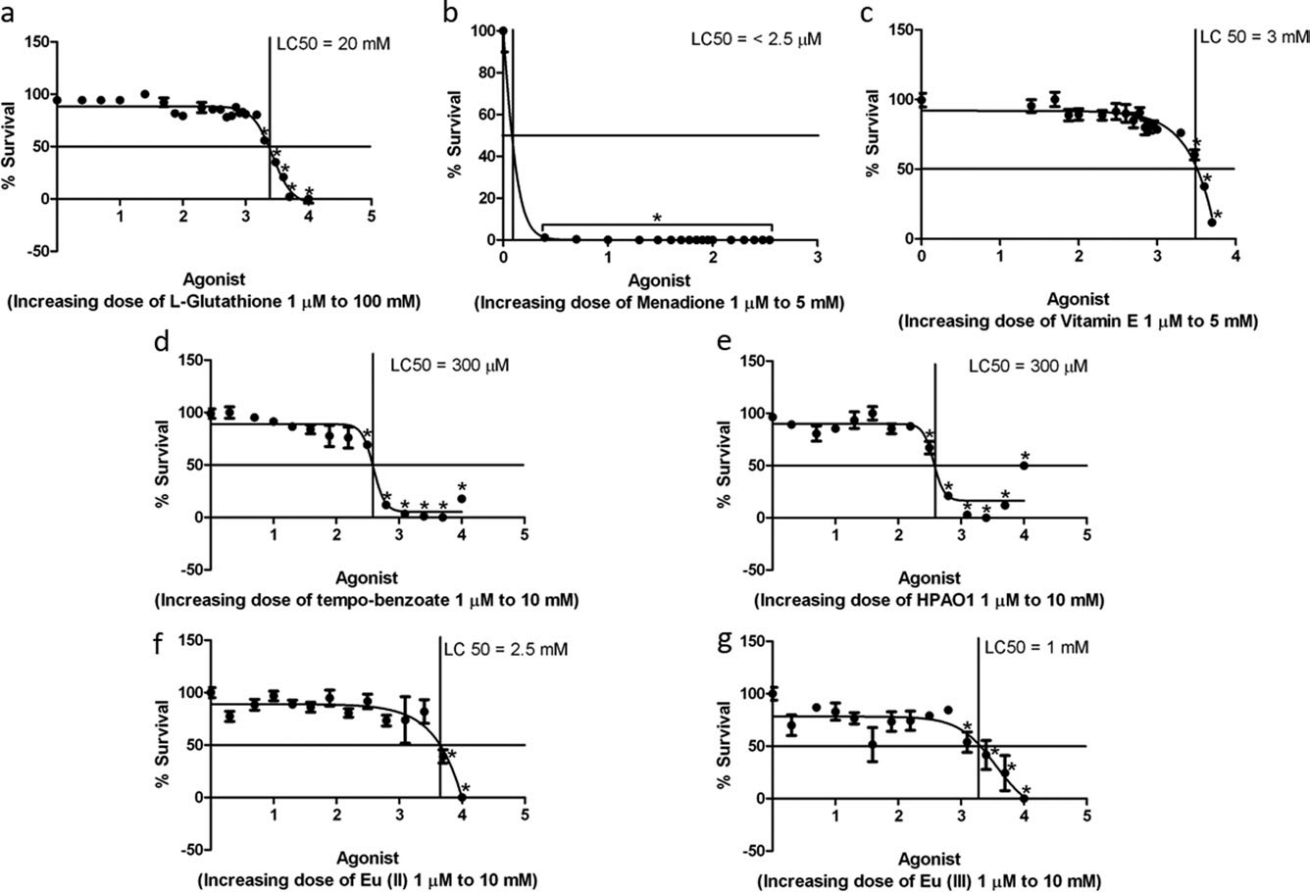
Dose response curves in PBMNC cells. The effect of increasing doses of l-Glutathione (panel a), Menadione (panel b), Vitamin E (panel c), Tempo (panel d), HPAO1 (panel e), Eu(II) (panel f), and Eu(III) (panel g) on PBMNC cell viability was investigated. Cell viability was determined using the ATPLite™ assay (Perkin-Elmer). Data were fitted to a sigmoidal dose response curve by log transforming the X column values then normalizing the Y column values. A non-linear regression was then performed before fitting the data to a sigmoidal dose response curve with a variable slope.

Negative (l-glutathione 1 mM) and positive (menadione 75 μm) controls were included in this study. In PBMNCs, the LC50 for l-glutathione was 20 mM and for menadione was <2.5 μM [Figure 2(a,b)]. Vitamin E, europium (II) chloride, and europium (III) chloride (diluted in RPMI1640 cell culture media) only adversely affected cell viability at relatively high concentrations (mM) in PBMNCs, the LC50 being 3 mM, 2.5 mM and 1 mM, respectively [Figure 2(c,f,g)]. TEMPO (diluted in ethanol) and HPAO1, adversely affected cells at relatively low doses, with their LC50 values both being 300 μM [Figure 2(d,e)]. A control for ethanol was included and the cytotoxic point for ethanol in PBMNCs cells was found to be at 10% v/v. The cytotoxic doses of 100 μM (HPAO1) and 200 μM (TEMPO) contained ethanol concentrations of 1.6% (v/v) and 3.1% (v/v), respectively, which was well below the cytotoxic level of ethanol.

### Anti-inflammatory effects of antioxidant compounds in U937 cells

**FIGURE 3:**
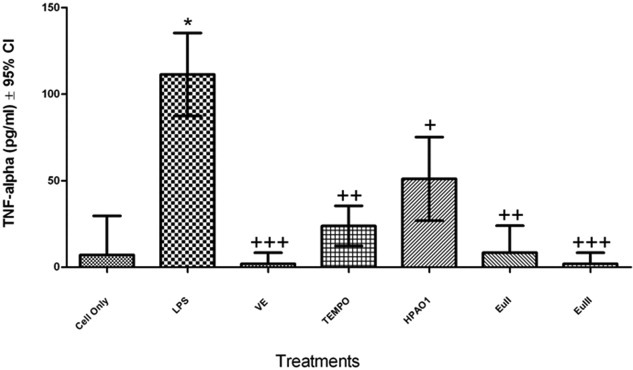
Effects of antioxidant compounds on TNF-α release by U937 cells stimulated with LPS. Treatments were as follows; LPS at time 0 followed by antioxidants at +3 h. Results show mean ± 95% confidence limits. * indicates a statistically significant difference compared to cells only negative control; + indicates significant reduction in LPS-stimulated TNF-α release after addition of antioxidant compounds (*p* < 0.05; ANOVA); and +++ indicates significant reduction in LPS-stimulated TNF-α release after addition of antioxidant compounds (*p* < 0.01; ANOVA).

U937 cells were stimulated with LPS for 3 h before the addition of antioxidant compounds. Antioxidants were then added at a non-cytotoxic dose to see if the antioxidant had an effect on the amount of the inflammatory cytokine TNF-α produced by the LPS-stimulated cells (see Materials and Methods). After an incubation period of 24 h, cell viability was determined by ATPlite™ assay. Cell viability was not adversely affected by any of the treatments when compared with a cell only control, with the exception of the menadione positive control [Figure 5, Supporting Information (panel a)]. The addition of LPS to U937 cells caused significant elevation of TNF-α release (Figure 3, *p* < 0.05 ANOVA). Addition of all the antioxidant compounds caused a significant decrease in the amount of TNF-α release, although the effect of HPAO1 was significantly lower than that of vitamin E, europium (II) chloride, and europium (III) chloride (Figure 3, *p* < 0.05 ANOVA).

### Anti-inflammatory effects of antioxidant compounds in PBMNCs

**FIGURE 4:**
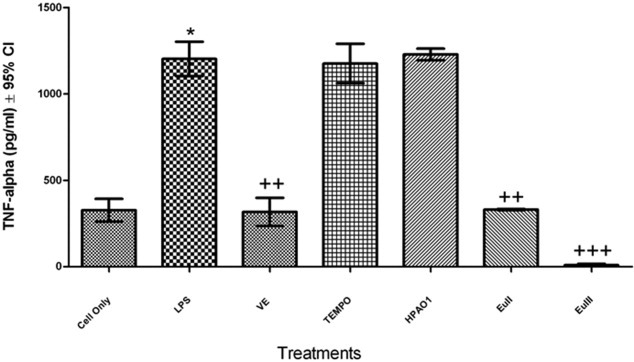
Effects of antioxidant compounds on TNF-α release by PBMNC cells stimulated with LPS. Treatments were as follows; LPS at time 0 followed by antioxidants at +3 h. Results show mean ± 95% confidence limits, * indicates a statistically significant difference compared to cells only negative control; + indicates significant reduction in LPS-stimulated TNF-α release after addition of antioxidant compounds (*p* < 0.05; ANOVA); and +++ indicates significant reduction in LPS-stimulated TNF-α release after addition of antioxidant compounds (*p* < 0.01; ANOVA).

PBMNCs were stimulated with LPS 3 h before the addition of antioxidant compounds. Antioxidants were added at a non-cytotoxic dose to see if the antioxidant had an effect on the production of the inflammatory cytokine TNF-α produced by the LPS-stimulated cells. After an incubation period of 24 h, cell viability was determined by ATPlite assay. Cell viability was not adversely affected by any of the treatments when compared to the cell only control, with the exception of the menadione positive control (see supplementary data, panel b). The addition of LPS to PBMNCs caused significant elevation of TNF-α release (Figure 4, *p* < 0.05 ANOVA). Addition of the antioxidant compounds vitamin E, europium (II) chloride, and europium (III) chloride caused a significant decrease in the amount of TNF-α release (Figure 4, *p* < 0.05 ANOVA). Addition of HPAO1 and TEMPO did not have any anti-inflammatory effects and did not significantly reduce the amount of TNF-α release (Figure 4).

## DISCUSSION

This study has highlighted the variability in cytotoxic and anti-inflammatory effects of a variety of compounds loosely associated under the umbrella term “antioxidants.” We have also highlighted the difference in sensitivity between different types of cells to these compounds. The U937 cell line was generally much less sensitive to any form of stimulation compared to the primary PBMNCs. This has been documented previously by comparing U937 cells to murine macrophages. The murine macrophages were more sensitive in terms of response to LPS stimulation and release on TNF-α than the U937 cells.^24^ Indeed, this study has shown that the U937 cells required a concentration of 1000 ng.ml^−1^ of LPS to stimulate the release of TNF-α, whereas the PBMNCs only required a concentration of 200 ng.ml^−1^. Also although the overall trend was similar between cell types in terms of TNF-α release, the actual amounts released were much higher in PBMNCs (>1000 pg/ml) than in U937 cells (<150 pg/ml). In the U937 cells, all the antioxidant compounds tested showed an anti-inflammatory effect, observed as a significant decrease in the amount of TNF-α released after LPS stimulation and incubation with the antioxidant compounds, although HPAO1 showed less effect than TEMPO, vitamin E, europium (II) chloride, and europium (III) chloride. In PBMNCs, addition of the antioxidant compounds vitamin E, europium (II) chloride, and europium (III) chloride caused a significant decrease in the amount of TNF-α release from LPS-stimulated cells, and therefore these compounds had a marked anti-inflammatory effect on pre-stimulated cells. The addition of HPAO1 and TEMPO did not have any anti-inflammatory effects, because these compounds did not significantly reduce the amount of TNF-α released from pre-stimulated cells.

In the area of TJR, it should be pointed out that currently the concentrations or doses of antioxidant compounds being added experimentally to UHMWPE are based on oxidative degradation protection of the polymer^25^ and measurement of the oxidative index after inclusion of antioxidant compounds at various doses.^9^, ^26^–^29^ Doses chosen (generally 1000 ppm to 3000 ppm) are based on efficiency of resistance to oxidation and are not currently based on any biological consideration.^9^, ^29^ Clearly although we want to add antioxidant compounds at doses that will provide oxidative resistance, we also need to be mindful that we do not add these compounds at potentially cytotoxic doses. We have previously shown that vitamin E is an antioxidant which when added to UHMWPE at 1000 ppm does not have any cytotoxic effects^30^ and indeed has been shown to be an effective anti-inflammatory.^23^

The current study has shown that lanthanides (europium II and II) are very effective anti-inflammatory compounds and only showed cytotoxicity in one of the conditions tested (eu(II) in U937 cells). Their anti-inflammatory activity was even greater than that observed with vitamin E. Potentially, these compounds could be added to UHMWPE and have the potential to reduce the osteolytic potential of UHMWPE wear particles containing them. TEMPO and HPAO1 showed no anti-inflammatory effects at all in PBMNCs and reduced anti-inflammatory effects in U937 cells. The results in PBMNCs suggest that caution should be used in considering such compound as suitable additives to UHMWPE. TEMPO and HPAO1 may indeed function as antioxidants reducing free radical damage to the polymer and increasing wear resistance of the polymer,^5^, ^10^ however, their biological effects may be significant in terms of their cytotoxicity. It is not known whether these chemicals would leach from UHMWPE TJR components *in vivo* and therefore pose a cytotoxic risk. Previous studies on vitamin E and HPAO1 have indicated that the compounds are not lost from the bulk material.^25^ However, it is not known whether the compounds will be lost from particulate wear debris that has a comparatively large surface area. Vitamin E is already in clinical use, reviewed in Ref.^31^, and may set the “gold standard” for antioxidant and anti-inflammatory effects.

In conclusion, this study has shown that many novel compounds were cytotoxic to U937 cells and PBMNCs, at relatively low concentrations (micromolar), specifically hindered phenol 3,5-di-tert-butyl-4-hydroxyhydrocinnamate (HPAO1), and TEMPO. Lanthanides were only cytotoxic at very high concentrations and were well tolerated by the cells at lower concentrations. Cytotoxic compounds also showed reduced anti-inflammatory effects, particularly in PBMNCs. Careful consideration should therefore be given to the use of any of these compounds as potential additives to UHMWPE.
